# Zinc hydroxychloride supplementation improves tibia bone development and intestinal health of broiler chickens

**DOI:** 10.1016/j.psj.2021.101254

**Published:** 2021-05-13

**Authors:** H.T.T. Nguyen, N. Morgan, J.R. Roberts, S.-B. Wu, R.A. Swick, M. Toghyani

**Affiliations:** ⁎ Department of Animal Science, School of Environmental and Rural Science, University of New England, Armidale, NSW 2351, Australia; † School of Life and Environmental Sciences, Faculty of Science, The University of Sydney, Sydney, NSW 2006, Australia

**Keywords:** zinc hydroxychloride, tibia trait, intestinal integrity, microbiota, gene expression

## Abstract

This study was conducted to investigate the effects of zinc (**Zn**), as a combination of oxide (ZnO) and sulfate (ZnSO_4_), compared with incremental levels of zinc hydroxychloride (**ZH**) on tibia traits, intestinal integrity, expression of selected jejunal genes, cecal short chain fatty acids and microbial composition in broilers. Day-old male Ross 308 chicks (*n* = 784) were randomly allocated to seven dietary treatments, each replicated seven times with 16 chicks per replication. The dietary treatments included a negative control diet (**NC**) with no supplemental Zn, a positive control (**PC**) with 100 mg/kg supplemental Zn from an ionic bound source combination (50 mg/kg ZnO + 50 mg/kg ZnSO_4_), and the NC diet supplemented with one of 20, 40, 60, 80, or 100 mg/kg Zn as ZH. The diets were fed over starter (1–14 d) and grower (14–35 d) phases, with tissue and digesta samples collected from 3 birds per replicate on days 14 and 35. The results showed that dietary Zn level had a significant effect on tibia breaking strength on d 35 (*P* < 0.05), and tibia Zn concentration both on d 14 and d 35 (*P* < 0.01). Dietary Zn levels linearly (*P* < 0.01) increased cecal lactic acid production, increased *Lactobacillus*, and decreased *Bacillus* and total bacteria counts (*P* < 0.05). Inclusion of 80 and 100 mg/kg Zn as ZH tended to upregulate the expression of claudin-1 (*P* = 0.088) and tight junction protein-1 (*P* = 0.086). The results obtained in this study suggest that a non-Zn supplemented diet can negatively influence tibia development and gut microbiota composition in broiler chickens. Higher supplemental Zn in the diet alters cecal microbiota population in favor of *Lactobacillus* and can decrease the total bacterial load. Supplemental Zn level in the feed have the potential to manipulate the jejunal gut integrity at a molecular level.

## INTRODUCTION

Zinc (**Zn**), as a trace mineral, performs various physiological roles in many biological processes in the body, all of which are essential for optimal growth and development (Park et al., 2004). The primary role of Zn in the body appears to be related to its association with enzymes and proteins, both as part of their molecular structure and by acting as co-enzymes and activators (Sarvari et al., 2015). The Zn requirement for broilers has been established at 40 mg/kg by the National Research Council (NRC, 1994). However, over past decades, the Zn requirement for broilers recommended by the major breeding companies has far exceeded the NRC recommendations, at up to 110 mg/kg added Zn for a Ross 308 broiler (Aviagen, 2014), or 100 mg/kg supplemental Zn for a Cobb 500 broiler (Cobb-Vantress, 2018). In a commercial production setting it is common practice to formulate diets to contain 100 to 120 mg/kg supplemental Zn (Feng et al., 2010).

Historically, Zn is supplied in the form of inorganic salts, such as sulfate (**ZnSO_4_**) and oxide (**ZnO**), in poultry diets to meet requirements. The ionic bonds in inorganic salts are very weak, allowing the metal ion to completely disassociate from the sulfate or oxide molecule once in contact with water (Miles et al., 1998). The disassociated molecule frees the Zn ion, which can then bind and antagonize a large number of dietary components such as other minerals, vitamins, enzymes or phytate molecules, impairing not only the absorption of Zn but also other minerals and nutrients (Underwood and Suttle, 1999). Zinc sulfate is highly water soluble, leading to the breakdown of vitamins and oxidation of fats, and rapid interactions with other feed components (Batal et al., 2001), whereas, ZnO is less reactive but also less bioavailable for poultry than feed-grade Zn sulfate (Edwards and Baker, 1999). Hence, using a combination of Zn sulfate and Zn oxide supplemented in broiler diets is common industry practice as it may increase Zn availability and lessen any negative effects of high sulfate.

Zinc hydroxychloride (**ZH**) is formed by covalent bonds between the Zn atom, multiple hydroxy groups and chloride ions, creating a stronger chemical bond compared to the ionic forms (sulfate and oxide). As a result of this crystallized structure, ZH is less water soluble than the ionic forms, and therefore, has a reduced likelihood of antagonistic reactions both in the feed and the upper digestive tract (Cromwell et al., 1998).

Commercially, the poultry industry is facing an increased incidence of skeletal disorders in broiler chickens, particularly leg problems, such as lameness and tibial dyschondroplasia. This could be partly due to deficiencies of some trace minerals (Lilburn, 1994). These problems may persist even when higher doses of trace minerals are included in the feed and premix, likely due to poor bioavailability and nutrient antagonistic interactions. It has long ago been documented that supplemental Zn mitigates leg disorders in broilers (Underwood, 1977). Research studies have shown that Zn administration positively affects bone formation (Seo et al., 2010), namely through its direct impacts on protein synthesis (Cowin, 2001; Scrimgeour et al., 2007), and its activity as hormonal growth mediators, such as influencing insulin-like growth factor I on osteoblasts (Wang et al., 2002).

The role of gut health is pivotal in broiler performance from hatch to the point of harvest (Shannon and Hill, 2019). Many studies have investigated the importance of Zn in gastrointestinal functionality and health. Zinc deficiency has been reported to negatively affect gut integrity by compromising the intestinal permeability (Crane et al., 2007; Zhang and Guo, 2009; Li et al., 2015), epithelial tissue integrity (Vallee and Falchuk, 1993) and the structure and function of the intestinal barriers (Rodriguez et al., 1996; Lambert et al., 2004). Body Zn status has also been shown to influence the intestinal microbiota community (Starke et al., 2014; Shannon and Hill, 2019), and regulation of the expression levels of several genes and proteins in the gut (Finamore et al., 2008; Zhang and Guo, 2009). However, the impact of ZH as an inorganic source of Zn on gut health has not been fully explored and studied particularly in modern broiler chickens. Thus, the present study was designed to investigate the effects of different levels of ZH inclusion compared with the ionic forms (ZnO and ZnSO_4_) on tibia characteristics and intestinal health status of broiler chickens, including the expression of selected jejunal tight junction genes, cecal short-chain fatty acid and microbiota composition.

## MATERIALS AND METHODS

All the experimental procedures applied in this study were reviewed and approved by the University of New England Animal Ethics Committee.

### Experimental Animal, Design, and Diets

A total of 784 male day-old Ross 308 broiler chicks were brought to the Centre of Animal Research and Training at the University of New England from a commercial hatchery (Darwalla Poultry Distributors Pty Ltd., Mount Cotton, Queensland, Australia). Chicks were weighed and assigned to seven dietary treatments based on a completely randomized design. Each treatment was replicated 7 times in floor pens, with 16 chicks per replicate (pen bodyweight of 720 ± 15 g).

Basal wheat-soybean meal diets were formulated to meet or exceed the requirements for starter (0–14 d) and grower (14–35 d) phases (Aviagen, 2014, Table 1), using a Zn-free mineral premix, serving as the negative control (**NC**) diets. For the positive control (**PC**) diets, the NC diet was supplemented with 50 mg/kg Zn as ZnO plus 50 mg/kg Zn as ZnSO_4_. The remaining 5 dietary treatments were the basal diet supplemented with one of 20, 40, 60, 80, or 100 mg/kg of Zn as Zn hydroxychloride (Selko IntelliBond Zn, Trouw Nutrition, Netherlands). The diets were pelleted, and the starter was crumbled to maximize intake. The room temperature was maintained at 34 ± 1.0^°^C during the first 3 d and then gradually reduced to 23^°^C at the end of wk 3. The lighting program and ventilation followed the recommendations set in the Ross 308 management guide (Aviagen, 2014). Birds had ad libitum access to water and feed throughout the entire study.

**Table 1:** Composition and nutritive value of the experimental diets (as-fed basis).

Ingredients %	Starter	Grower
Wheat	56.32	59.60
Soybean meal dehulled	29.70	23.00
Canola meal	5.63	6.41
Rice bran	3.87	5.23
Canola oil	2.00	3.38
Limestone	1.17	1.17
Dicalcium phosphate^1^	0.38	0.19
Sodium chloride	0.17	0.13
Sodium bicarbonate	0.12	0.13
Mineral premix^2^	0.10	0.10
Vitamin premix^3^	0.09	0.09
Choline Cl 60%	0.06	0.06
L-lysine	0.20	0.22
D,L-methionine	0.21	0.19
L-threonine	0.05	0.07
Xylanase	0.02	0.02
Phytase	0.01	0.01
Total	100	100
*Calculated nutrients (analyzed values)*		
ME, kcal/kg	3,000	3,140
Crude protein %	23.96 (24.53)	21.71 (22.21)
Crude fat %	4.42	6.06
Crude fiber %	3.18	3.17
d Arg %	1.33	1.15
d Lys %	1.24	1.10
d Met %	0.53	0.49
d M+C %	0.90	0.83
Calcium %	0.85 (0.98)	0.80 (0.88)
Total phosphorous %	0.55 (0.58)	0.51 (0.55)
Phosphorus avail %	0.43	0.40
Sodium %	0.17	0.16
Chloride %	0.20	0.17
Choline mg/kg	1,600	1,500
Linoleic 18:2 %	1.32	1.71

1 Dicalcium phosphate contained: phosphorus, 18%; calcium, 21%.

2 The Zn-free trace mineral concentrate supplied per kilogram of diet: Cu (sulfate), 16 mg; Fe (sulfate), 40 mg; I (iodide), 1.25 mg; Se (selenate), 0.3 mg; Mn (sulfate and oxide), 120 mg; cereal-based carrier, 128 mg; mineral oil, 3.75 mg.

3 Vitamin concentrate supplied per kilogram of diet: retinol, 12,000 IU; cholecalciferol, 5,000 IU; tocopheryl acetate, 75 mg, menadione, 3 mg; thiamine, 3 mg; riboflavin, 8 mg; niacin, 55 mg; pantothenate, 13 mg; pyridoxine, 5 mg; folate, 2 mg; cyanocobalamin, 16 μg; biotin, 200 μg; cereal-based carrier, 149 mg; mineral oil, 2.5 mg.

### Sample Collection

Triple representative composite samples from all the diets and premixes were collected and analyzed to determine Zn concentration, each determined in duplicate (Table 2).

**Table 2:** The analyzed Zn content of mineral premixes and diets.^1^

			Calculated Zn mg/kg		Analyzed Zn mg/kg	
Treatments^2^	Supplemental Zn mg/kg	Premix g/ kg	Starter	Grower	Stater	Grower
PC	100	102.70	130	140	127	138
NC	0	0.28	30	40	31	43
ZH 20 mg/kg	20	21.10	50	60	51	61
ZH 40 mg/kg	40	43.50	70	80	73	78
ZH 60 mg/kg	60	63.30	90	100	96	98
ZH 80 mg/kg	80	91.40	110	120	113	135
ZH 100 mg/kg	100	102.10	130	140	135	156

1 Values based on chemical analysis of duplicate samples of each premix and diet, reported on an as-fed basis.

2 PC: Positive control, 100 mg/kg Zn supplied in form of ZnO and ZnSO_4_; NC: Negative control, no added Zn; ZH: Zinc hydroxychloride (Intellibond Zn).

On d 14 of the experiment, 3 birds per replicate were euthanized and cecal content from each individual bird was collected into ice-cooled containers and homogenized, and then subsequently frozen at −20°C for measurement of short chain fatty acids (**SCFAs**). Subsamples of cecal digesta were collected and stored in Eppendorf tubes and directly snap-frozen in liquid nitrogen and kept at −80°C until analysis for microbial population by real-time quantitative PCR (**qPCR**). From those three birds, a 1-cm section of jejunum from each bird was collected at the Meckel's diverticulum. The jejunum sections were flushed with ice-cold phosphate buffered saline solution (pH 7.4) and transferred into 2 mL Eppendorf tubes with 1.5 mL RNAlater (Qiagen, Hilden, Germany), and then stored in a −80°C freezer prior to gene expression analysis.

### Tibia Traits and Mineral Analysis in Tibia Ash and Diets

Right tibias were collected on both d 14 and 35 of the study, from 3 birds, individually. The length and width of the tibias were measured using a digital caliper. The tibias were subjected to breaking strength measurement using an Instron instrument (LX 300 Instron Universal Testing Machine, Instron Corp., Norwood, MA). The tibia bone samples were then dried and ashed (Carbolite, Sheffield, UK) at 600°C for 6 h. Moisture-free tibia ash was expressed as the percentage of tibia ash relative to dry tibia weight. The mineral content of the tibia ash and diet samples were determined by inductively coupled plasma-optical emission spectrometer (Agilent, Mulgrave, Victoria, Australia).

### Cecal SCFAs Analysis

Cecal concentrations of SCFAs were measured according to the method described by Jensen et al. (1995) with minor modifications. Briefly, around 0.8 g of cecal digesta was weighed into centrifuge tubes and 1 mL of 0.01M ethyl butyric acid (internal standard) solution added. The solution was vortexed and centrifuged at 15,000 × *g* at 5°C for 20 min. Then 1-mL supernatant was transferred to 8 mL vials (placed on ice), then 2.5 mL of ether and 0.5 mL of concentrated HCl (36%) were added. The solution was vortexed for one min, then centrifuged at 3,000 × *g* for 15 min in 5°C; 400 µL of the resulting supernatant was transferred into 2 mL gas chromatograph vials and mixed with 40 µL N-tert-butyl-dimethylsilyl-N-methyltrifluoroacetamide. This solution was vortexed and heated at 80°C in a heating block for 20 min and then left at room temperature for at least 48 h. Then 0.5 mL ether was added into the gas chromatograph vials before analysis using a Varian CP3400 CX gas Chromatograph (Varian Analytical Instruments, Palo Alto, CA). The value of SCFAs is expressed as μmol/g wet cecal digesta.

### Quantification of Cecal Bacterial Groups

Cecal bacterial DNA extraction was performed following the method described by Kheravii et al. (2018). In brief, around 60 mg of frozen cecal samples were added to 300 mg of glass beads. QIAxtractor DNA Reagents and QIAxtractor DNA plasticware kits (Qiagen, Inc., Doncaster, VIC, Australia) were used for the DNA extraction. Then samples were lysed with 300 µL of Qiagen Lysis Buffer, with cells disrupted by shaking the tubes in a bead beater mill (Retsch GmbH & Co, Haan, Germany). Samples were then placed in a heating block for 2 h at 55°C followed by centrifugation at 20,000 × *g* for 5 min. Then the DNA was extracted using an X-tractor gene automated DNA extraction system (Corbett Life Science, Sydney, Australia). The extracted DNA samples were checked for quantity and purity on a NanoDrop ND-8000 spectrophotometer (Thermo Fisher Scientific). DNA with ratios of A260/A280 being > 1.8 were considered of high purity and were stored at −20°C.

The extracted cecal DNA was diluted 20 times in nuclease-free water and the qPCR was performed to quantify 6 bacterial groups with a real-time PCR system Rotorgene 6000 (Corbett, Sydney, Australia). The PCR was performed in duplicate for each sample. A SYBRGreen containing Mix (SensiMix SYBR No-Rox, Bioline, Sydney, Australia) was applied for all groups of bacteria to quantify total bacteria, *Bacillus, Bacteroides, Bifidobacterium, Lactobacillus*, and Enterobacteriaceae. The primers used for these bacterial groups are shown in Table 3. Bacteria numbers were expressed as log_10_ (genomic DNA copy number)/g wet digesta.

**Table 3:** Primer sequences used for the qPCR analysis of selected bacteria groups.

Target group	Primer sequences (5ʹ-3ʹ)	Annealing temp. (C^o^)	Reference
*Bacillus* spp.	F- GCA ACG AGC GCA ACC CTT GA R- TCA TCC CCA CCT TCC CC GGT	63	(Zhang et al., 2015)
*Bacteroides* spp.	F-GAG AGG AAG GTC CCC CAC R-CGC TAC TTG GCT GGT TCA G	63	(Layton et al., 2006)
*Bifidobacterium* spp.	F-GCG TCC GCT GTG GGC R-CTT CTC CGG CAT GGT GTT G	63	(Requena et al., 2002)
Enterobacteriaceae	F-CAT TGA CGT TAC CCG CAG AAG AAG C R-CTC TAC GAG ACT CAA GCT TGC	63	(Bartosch et al., 2004)
*Lactobacillus* spp.	F-CAC CGC TAC ACA TGG AG R-AGC AGT AGG GAA TCT TCC A	63	(Wise and Siragusa, 2007)
Total bacteria	F-CGG YCC AGA CTC CTA CGG G R-TTA CCG CGG CTG CTG GCA C	63	(Lee et al., 1996)

### Jejunal Gene Expression Analysis

Total RNA from approximately 80 mg of jejunal tissues was extracted after homogenization in TRIsure (Bioline, Sydney, Australia), following the manufacturer's instructions. Total RNA of each sample was purified using ISOLATE II RNA Mini Kit (Bioline, Sydney, Australia) as per the manufacturer's instructions. The quantity and quality of total RNA were determined using a NanoDrop ND-8000 spectrophotometer (Thermo Fisher Scientific, Waltham, MA). RNA integrity number (RIN) was evaluated with an Agilent 2100 Bioanalyzer (Agilent Technologies, Inc., Waldbronn, Germany) using RNA 6000 Nano kit. RNA samples were considered of high quality for downstream analysis if the RIN value was greater than 7.5 (Fleige et al., 2006).

The extracted RNA of each sample was reverse transcribed into cDNA using the SensiFAST cDNA Synthesis Kit as per the manufacturer's instructions. The cDNA samples were diluted 1:10 with nuclease-free water and stored at −20˚C for further analysis.

qPCR was performed using a SYBR Green kit SensiFAST SYB No-ROX (Bioline, Sydney, Australia) with Rotorgene 6000 real-time PCR machine (Corbett Research, Sydney, Australia). The geNorm module in qbase+ software (Biogazelle, Zwijnaarde, Belgium) was employed to determine two most stable genes among eight different reference genes, 18S, ACTB, GAPDH, HPRT1, HMBS, TBP, SDHA, and YWHAZ. Based on the expression stability, ACTB and HMBS were used to normalize the target genes in the jejunum. The primers of the selected genes are described in Table 4.

**Table 4:** Primers used for quantitative real-time PCR.

Gene symbol	Gene name	Primer sequence (5ʹ-3ʹ)	Ta	Size (bp)	References
CLDN1	Claudin 1	F-CTTCATCATTGCAGGTCTGTCAG R-AAATCTGGTGTTAACGGGTGTG	60	103	Gharib-Naseri et al. (2020)
CLDN5	Claudin 5	F-GCAGGTCGCCAGAGATACAG R-CCACGAAGCCTCTCATAGCC	60	162	Gharib-Naseri et al. (2020)
JAM2	Junctional adhesion molecule-2	F-AGACAGGAACAGGCAGTGCTAG R-ATCCAATCCCATTTGAGGCTAC	60	135	Gharib-Naseri et al. (2020)
OCLD	Occludin	F- ACGGCAGCACCTACCTCAA R- GGGCGAAGAAGCAGATGAG	60	123	Du et al. (2016)
TJP1	Tight junction protein 1	F-GGATGTTTATTTGGGCGGC R-GTCACCGTGTGTTGTTCCCAT	60	187	Gharib-Naseri et al. (2020)

### Statistical Analysis

All the data derived were checked for normal distribution prior to conducting statistical analysis, and then analyzed as one-way ANOVA using General Linear Model procedure of the Statistical Analysis System (SAS 9.3 package) (SAS Institute Inc., 2010). Each single pen was considered as an experimental unit and the values presented in the tables are means with pooled standard error of the mean (SEM) (*n* = 49). When a significant effect of treatment was detected (*P* ≤ 0.05), the means were separated by Tukey's test. The linearity of responses to dietary ZH levels were established using linear and quadratic regression.

## RESULTS

### Mineral Concentration in Diets and Tibia Bone Traits

The mean analyzed Zn concentration in the basal diet for the starter phase was 31 mg/kg and 43 mg/kg for the grower phase (Table 2). The tibia characteristics and mineral content determined on d 14 and 35 are presented in Table 5. The dietary treatments did not affect tibia breaking strength on d 14 (*P* > 0.05). However, there was a significant effect of Zn supplementation on tibia breaking strength on d 35, where birds fed ZH 100 mg/kg had higher tibia breaking strength than those fed the NC and ZH 20 mg/kg diets (*P* < 0.05). Tibia ash percentage either on d 14 or 35, as well as length and width on d 35, were not influenced by the dietary treatments (*P* > 0.05). Broilers fed the NC diet had significantly lower (*P* < 0.01) tibia Zn concentration compared to those fed the other diets on d 14. On d 35, supplementation with ZH 100 mg/kg resulted in more Zn being deposited in the tibia than the NC and ZH 20 mg/kg (*P* < 0.01). Zinc accumulation in the tibia responded linearly (*P* < 0.01) to supplemental Zn as ZH on d 14 and 35, where Zn deposition increased as ZH dose increased. Tibia calcium and phosphorous content were not statistically affected by Zn sources and levels on d 14 or 35 (*P* > 0.05).

**Table 5:** Tibia characteristics and mineral concentration of broilers in response to the dietary treatments.

	Breaking strength (N/mm^2^)		Measurements D 35 (mm)		Ash (%)		Zinc (µg/g)		Calcium (%)		Phosphorous (%)	
Treatments^1^	D 14	D 35	Length	Width	D 14	D 35	D 14	D 35	D 14	D 35	D 14	D 35
PC	118.4	386^ab^	93.4	8.11	48.9	45.1	436^a^	328^ab^	29.4	23.6	17.8	16.2
NC	112.9	367^b^	92.1	7.92	48.6	44.7	367^b^	310^bc^	29.2	23.3	17.9	16.1
ZH 20 mg/kg	111.9	368^b^	92.1	8.04	48.4	44.8	427^a^	304^c^	29.2	23.3	17.9	16.1
ZH 40 mg/kg	111.9	387^ab^	91.6	8.02	48.3	43.6	425^a^	317^abc^	29.3	23.6	18.0	16.3
ZH 60 mg/kg	109.3	388^ab^	92.2	7.87	48.2	44.7	435^a^	321^abc^	29.1	23.5	17.8	16.1
ZH 80 mg/kg	117.8	389^ab^	94.3	8.06	49.4	44.2	431^a^	326^ab^	29.1	23.6	17.8	16.0
ZH 100 mg/kg	114.1	426^a^	92.7	8.31	48.5	44.9	450^a^	332^a^	29.2	23.8	18.0	16.1
SEM	3.47	11.25	0.641	0.119	0.351	0.397	6.40	4.64	0.568	0.165	0.312	0.114
*ANOVA P*- value	0.516	0.014	0.081	0.237	0.211	0.202	0.001	0.005	0.351	0.237	0.990	0.518
Linear	0.139	0.002	0.031	0.064	0.242	0.390	<0.001	<0.001	0.605	0.113	0.843	0.825
Quadratic	0.238	0.008	0.092	0.103	0.400	0.129	0.006	<0.001	0.665	0.481	0.969	0.976

^a–c^Values in a column with no common superscripts differ significantly (*P* ≤ 0.05); NS: not significant (*P* > 0.05).

Mean values are based on 3 birds per replicate and 7 replicates per treatment.

1 PC: Positive control, 100 mg/kg Zinc supplied in form of ZnO and ZnSO_4_; NC: Negative control, no added Zinc; ZH: Zinc hydroxychloride (Intellibond Zn).

### Cecal Bacterial Groups, SCFAs, and Jejunal Gene Expression

There was no significant effect of dietary treatments on cecal bacterial groups on d 14 (*P* > 0.1; Table 6), but examination of polynomial trends, *Lactobacillus* count linearly increased (*P* < 0.05) when dietary ZH content increased. *Bacillus* and total bacteria counts were also linearly affected by Zn content of the diets, and their counts decreased as supplemental ZH increased (*P* < 0.05).

**Table 6:** Bacterial composition (Log_10_ genomic DNA copy numbers g^−1^) in ceca content of broilers at d 14.

Treatments^1^	*Lactobacillus*	*Bacteriodes*	*Bacillus*	*Bifidobacterium*	Enterobacteriaceae	Total bacteria
PC	9.42	5.35	8.62	5.92	8.37	9.90
NC	9.32	5.98	8.89	6.18	8.55	9.94
ZH 20 mg/kg	9.33	6.12	8.78	5.86	8.45	9.93
ZH 40 mg/kg	9.41	5.92	8.84	6.37	8.73	9.94
ZH 60 mg/kg	9.51	6.05	8.97	6.13	8.65	9.92
ZH 80 mg/kg	9.51	5.98	8.61	6.53	9.19	9.91
ZH 100 mg/kg	9.53	6.36	8.46	6.17	8.88	9.87
SEM	0.082	0.532	0.130	0.212	0.250	0.023
*ANOVA P-values*	0.370	0.917	0.106	0.315	0.299	0.430
Linear	0.036	0.821	0.013	0.761	0.314	0.031
Quadratic	0.082	0.940	0.022	0.643	0.455	0.088

Mean values are based on 3 birds per replicate and 7 replicates per treatment; NS: not significant (*P* > 0.05).

1 PC: Positive control, 100 mg/kg Zinc supplied in form of ZnO and ZnSO_4_; NC: Negative control, no added Zinc; ZH: Zinc hydroxychloride (Intellibond Zn).

According to the data presented in Table 7, one-way ANOVA analysis did not show any significant differences (*P* > 0.1) among the dietary Zn treatments for SCFAs concentration in the ceca. However, the polynomial analysis revealed both linear (*P* < 0.01) and quadratic (*P* < 0.05) responses to dietary Zn inclusion on the concentration of lactic acid in the ceca, where increasing supplemental ZH increased lactic acid content of cecal digesta on d 14; the highest values were observed in the 100 mg/kg and 80 mg/kg ZH fed groups.

**Table 7:** Concentration of short chain fatty acids (μmol g^−1^ digesta) in ceca content of broilers at d 14.

Treatments^1^	Acetic	Propionic	Iso-butyric	Butyric	Iso-valeric	Valeric	Lactic	Succinic	Total
PC	126	7.54	1.42	34	0.59	1.58	1.87	21.4	195
NC	123	6.52	1.66	31	0.66	1.84	1.32	21.0	187
ZH 20 mg/kg	124	7.81	1.73	29	0.75	1.98	1.54	22.6	189
ZH 40 mg/kg	129	7.65	1.52	39	0.71	1.60	1.68	25.0	207
ZH 60 mg/kg	134	8.44	1.61	37	0.83	1.48	1.86	21.5	207
ZH 80 mg/kg	150	8.96	1.83	40	0.98	1.52	2.05	23.0	229
ZH 100 mg/kg	140	8.80	1.53	43	0.86	1.54	2.15	26.0	224
SEM	18.88	0.877	0.306	6.61	0.171	0.297	0.242	5.63	26.6
*ANOVA P-*values	0.943	0.482	0.973	0.736	0.734	0.873	0.221	0.994	0.887
Linear	0.428	0.106	0.664	0.186	0.568	0.226	0.006	0.775	0.309
Quadratic	0.669	0.143	0.876	0.371	0.601	0.421	0.027	0.950	0.528

Mean values are based on 3 birds per replicate and 7 replicates per treatment; NS: not significant (*P* > 0.05).

1 PC: Positive control, 100 mg/kg Zinc supplied in form of ZnO and ZnSO_4_; NC: Negative control, no added Zinc; ZH: Zinc hydroxychloride (Intellibond Zn).

The mRNA expression of 5 genes involved in gut integrity were investigated in response to Zn level and source (Table 8). The supplemental inclusion of 80 and 100 mg/kg Zn as ZH tended to upregulate the expression of claudin-1 (*P* = 0.088) and tight junction protein-1 (*P* = 0.086). Increasing dietary Zn supplementation both linearly (*P* < 0.05) and quadratically (*P* < 0.05) upregulated the expression of tight junction protein-1, with the highest values observed in 100 mg/kg and 80 mg/kg ZH fed groups.

**Table 8:** Effect of dietary treatments on expression of jejunal tight junction genes.^1^

Treatments^2^	CLDN1	CLDN5	JAM2	OCLD	TJP1
PC	0.95	1.12	1.04	0.96	1.18
NC	0.97	1.04	1.05	0.99	0.93
ZH 20 mg/kg	0.92	0.94	0.95	0.95	1.26
ZH 40 mg/kg	0.83	1.06	1.06	1.04	1.23
ZH 60 mg/kg	0.98	0.97	1.07	1.19	1.43
ZH 80 mg/kg	1.08	0.95	0.99	1.04	1.52
ZH 100 mg/kg	1.03	1.00	1.03	1.24	1.60
SEM	0.057	0.073	0.068	0.107	0.162
*ANOVA P*-values	0.088	0.564	0.875	0.345	0.086
Linear	0.151	0.737	0.901	0.254	0.020
Quadratic	0.262	0.598	0.992	0.469	0.027

Mean values are based on 3 birds per replicate and 7 replicates per treatment; NS: not significant (*P* > 0.05).

CLDN1: Claudin-1; CLDN5: Claudin-5; JAM2: Junctional adhesion molecule-2; OCLD: Occludin; TJP1: Tight junction protein-1.

1 The relative expression levels of the genes of respective treatment groups are expressed as means of normalized relative quantities (NRQ). Relative quantities for individual genes are scaled to the average across all unknown samples per target gene.

2 PC: Positive control, 100 mg/kg Zinc supplied in form of ZnO and ZnSO_4_; NC: Negative control, no added Zinc; ZH: Zinc hydroxychloride (Intellibond Zn).

## DISCUSSION

This study investigated the effects of dietary Zn source and level on the tibia bone traits, cecal SCFAs, cecal bacterial groups and jejunal gene expression of broilers fed wheat-soybean meal based diets. The Zn concentrations in the basal NC diet for the starter (31 mg/kg) and the grower (43 mg/kg) phases were close to the recommendation of NRC (1994). The supplemental Zn levels (0–100 mg/kg) in this study were selected to cover the established requirements of 40 mg/kg (NC diet) by the National Research Council (NRC, 1994), and the recommended level of 100 mg/kg of supplemental Zn by major breeding companies (Aviagen, 2014; Cobb-Vantress, 2018).

Leg health is of pivotal importance in meat chicken production as birds with leg problems are less likely to reach market body weight, due to difficulties in reaching the feeders and drinker lines, potentially resulting in increased incidences of breast blisters indirectly and off-grade carcasses during processing (Štofaníková et al., 2011). Bones function as a reserve of most of the trace minerals in broilers, including Zn; thus bone characteristics such as bone breaking strength, bone mineral concentration (Kim et al., 2006) and bone ash have often been used as sensitive indicators of bone status and response to both macro and trace mineral supplementation (Ma et al., 2018). Bone breaking strength is positively correlated with collagen crosslink content (Rath et al., 1999). Being an essential element for collagen formation, Zn deficiency can impair collagen synthesis, leading to decreased bone strength and mineralization (Rath et al., 2000). In addition, supplementation of Zn has been reported to enhance the anabolic effect of insulin-like growth factor I on osteoblasts (Wang et al., 2002), which directly impact bone development. The results obtained in this study indicate that tibia strength on day 35 responded linearly to the Zn concentration of the diet, and Zn supplied in form of ZH at 100 mg/kg resulted in stronger tibias (~10%) than the same concentration from ZnO and ZnSO_4_ combination. The fact that ZH is less reactive than ionic forms of Zn (Cao et al., 2000a), resulting in higher bioavailability and provision of Zn for collagen synthesis and osteoblast growth, could to some extent explain the improved tibia breaking strength in 100 mg/kg ZH group.

Tibia ash percentage is influenced to a greater extent by provision of Ca and available P and their ratios in the feed, as opposed to by the trace mineral profile of the feed. In line with our findings, there are other studies which also reported no significant effect of Zn source or level on tibia ash percentage (Mohanna and Nys, 1999; Olukosi et al., 2018). The low content of tibia Zn in the NC group is similar to findings presented by Henry et al. (1987), Huang et al. (2007) and Vieira et al. (2013), who reported the tibia Zn concentration was significantly decreased when birds were fed a nonsupplemented Zn diet. Both tibia Ca and P content were consistent across different treatments, implying that different sources and levels of supplemental Zn in this study did not interfere with the absorption and utilization of Ca and P in the bone.

Diet nutrient profile and digestibility play an important role in dictating the gut microbiota population (Apajalahti and Vienola, 2016; Dong et al., 2017), since compounds of dietary origin are the most important growth substrates for microbes. Zinc is an essential mineral for the growth of numerous bacteria, and a substantial relationship between dietary Zn content and the gut microbiota ecosystem has previously been reported by Shao et al. (2014). Yazdankhah et al. (2014) reported that the antimicrobial properties of Zn could alter gut microbiota communities, reducing fermentation loss of nutrients, and to some degree suppressing gut pathogens. A higher population of *Lactobacillus* bacteria can potentially prevent the colonization of pathogenic bacteria, through competitive exclusion and production of antimicrobial and anti-inflammatory agents (Fang, 2010). Likewise, Shao et al. (2014) reported a favorable effect of supplemental Zn on the number of *Lactobacillus* bacteria. The higher count of *Lactobacillus* could also have suppressed the *Bacillus* count, as both strains produce lactic acid and proliferate as competitors. The observed higher numbers of *Bifidobacterium* and Enterobacteriaceae in birds given 80 mg/kg Zn as ZH, compared to those fed 100 mg/kg supplemental Zn as ionic forms or ZH, may be due to the sensitivity of these bacterial groups to Zn levels in the feed.

SCFAs, the end-products of the gut microbiota following fermentation of complex carbohydrates in ceca, are a source of energy for animals. They are necessary for metabolism of the intestinal epithelial cells (Meimandipour et al., 2010), thus increasing the overall gastrointestinal absorption surface (Dibner and Richards, 2005). Lactic acid is the main product of carbohydrate fermentation performed by lactic acid bacteria, such as *Lactobacilli* and *Streptococci*, and is an energy source for bacterial synthesis of acetate, propionate, and butyrate (Janczyk et al., 2015). In this study, the concentration of lactic acid was linearly increased by increasing the supplemental Zn in the feed. This observation is in complete agreement with the positive linear response observed between cecal *Lactobacillus* count and dietary Zn concentration. Zinc is involved in carbohydrate metabolism (Salim et al., 2008); thus, lower SCFA concentration in the non-Zn birds may be due to decreased output of carbohydrate metabolism and fermentation, via changes in microbial metabolic pathway (Reed et al., 2015).

When gut permeability is compromised, microbial toxins and pathogens can pass in between the epithelial cells into the bloodstream, leading to cell damage or intestinal inflammation, decreased performance and increased mortality rate. Epithelial cells are bound to each other by complex protein structures known as tight junctions; thus, changes in the intestinal permeability may be influenced by modulation and functionality of tight junction proteins, in which bacterial-derived proteases may cause degradation by a broad range of mechanisms (Awad et al., 2017). Various proteins in tight junctions, including claudin and occludin proteins, regulate epithelial permeability, and contribute to the maintenance of the barrier integrity for the intestinal tracts of animals (Krause et al., 2008). According to Shao et al. (2017), dietary Zn supplementation enhances the intestinal epithelial barrier function by upregulating tight junction expression. In the present study, higher Zn supplementation tended to upregulate the expression of tight junction protein-1 and claudin-1 in the jejunum. Similarly, Zhang and Guo (2009) and Hu et al. (2013) also reported that dietary Zn supplementation increased the expression of tight junction proteins and claudin-1.

## CONCLUSIONS

In summary, the findings of this study suggest a non-Zn supplemented diet negatively affects optimum bone development and gut health in broiler chickens. Higher levels of supplemental Zn in the diet alter cecal microbiota population in favor of *Lactobacillus* and can decrease the total bacterial load. The higher tibia breaking strength, and upregulation of claudin-1 and tight junction protein-1 expression with 100 mg/kg Zn added in the form of ZH, compared to 100 mg/kg Zn from ionic forms (the combination of Zn oxide and Zn sulfate), suggests superior bioavailability of Zn from the hydroxychloride source when administered at a similar dose.
